# Genome-Wide Association Scan in HIV-1-Infected Individuals Identifying Variants Influencing Disease Course

**DOI:** 10.1371/journal.pone.0022208

**Published:** 2011-07-21

**Authors:** Daniëlle van Manen, Olivier Delaneau, Neeltje A. Kootstra, Brigitte D. Boeser-Nunnink, Sophie Limou, Sebastiaan M. Bol, Judith A. Burger, Aeilko H. Zwinderman, Perry D. Moerland, Ruben van 't Slot, Jean-François Zagury, Angélique B. van 't Wout, Hanneke Schuitemaker

**Affiliations:** 1 Department of Experimental Immunology, Sanquin Research, Landsteiner Laboratory, and Center for Infectious Diseases and Immunity Amsterdam (CINIMA) at the Academic Medical Center of the University of Amsterdam, Amsterdam, The Netherlands; 2 Chaire de Bioinformatique, Conservatoire National des Arts et Métiers and ANRS Genomic Group, Paris, France; 3 Department of Clinical Epidemiology, Biostatistics and Bioinformatics at the Academic Medical Center of the University of Amsterdam, Amsterdam, The Netherlands; 4 Netherlands Bioinformatics Center (NBIC), Nijmegen, The Netherlands; 5 Complex Genetics Section, Department of Biomedical Genetics at the University Medical Center, Utrecht, The Netherlands; Institut National de la Santé et de la Recherche Médicale, France

## Abstract

**Background:**

AIDS develops typically after 7–11 years of untreated HIV-1 infection, with extremes of very rapid disease progression (<2 years) and long-term non-progression (>15 years). To reveal additional host genetic factors that may impact on the clinical course of HIV-1 infection, we designed a genome-wide association study (GWAS) in 404 participants of the Amsterdam Cohort Studies on HIV-1 infection and AIDS.

**Methods:**

The association of SNP genotypes with the clinical course of HIV-1 infection was tested in Cox regression survival analyses using AIDS-diagnosis and AIDS-related death as endpoints.

**Results:**

Multiple, not previously identified SNPs, were identified to be strongly associated with disease progression after HIV-1 infection, albeit not genome-wide significant. However, three independent SNPs in the top ten associations between SNP genotypes and time between seroconversion and AIDS-diagnosis, and one from the top ten associations between SNP genotypes and time between seroconversion and AIDS-related death, had P-values smaller than 0.05 in the French Genomics of Resistance to Immunodeficiency Virus cohort on disease progression.

**Conclusions:**

Our study emphasizes that the use of different phenotypes in GWAS may be useful to unravel the full spectrum of host genetic factors that may be associated with the clinical course of HIV-1 infection.

## Introduction

The clinical course of HIV-1 infection can be highly variable between individuals. The period of asymptomatic disease after HIV-1 infection in the absence of antiviral therapy is typically 7–11 years [1], [2], with extremes of disease progression within 2 years, or virtually no disease progression for more than 15 years [3]. The genetic make-up of an individual has been shown to play a role in the susceptibility to HIV-1 infection and/or the rate of disease progression. Some of the observed variation could be attributed to human leukocyte antigen (HLA) types. In the Caucasian population, HLA-B5701 and HLA-B27 are most strongly associated with prolonged survival, whereas a variant of HLA-B35 is linked to an accelerated progression to AIDS [4]–[7]. Another well known example is the 32 base pair deletion in the gene coding for the chemokine receptor CCR5 that serves as a coreceptor for HIV-1. This polymorphism has been associated with reduced susceptibility to infection [8], [9] and a slower rate of disease progression [10]–[12]. However, all host genetic factors identified to date can explain the clinical course of HIV-1 infection in only a minority of individuals [13], [14].

In the last couple of years several genome-wide association studies (GWAS) have been published to reveal additional host genetic factors that are associated with HIV-1 control. Fellay et al published two single nucleotide polymorphisms (SNP) on chromosome 6, one located in HCP5 (rs2395029) and in high linkage disequilibrium (LD) with HLA-B57, and one at position -35 in the HLA-C gene region (rs9264942), to be associated with a lower viral load set-point [14], [15], which could be confirmed by us and others [16], [17]. Other GWAS confirmed the important role of the HLA region on chromosome 6 in the clinical course of HIV-1 infection, and found potentially interesting additional associations which need confirmation in other cohorts [18]–[23].

Although HIV-1 viral load is established as a good predictor for AIDS disease progression [24], [25], several studies have shown that it is not the sole determinant for variation in disease progression and CD4^+^ T-cell depletion [26]–[28]. To reveal additional host genetic factors that are associated with the clinical course of HIV-1 infection, we designed a GWAS in the Amsterdam Cohort Studies (ACS) on HIV-1 infection and AIDS and examined the association between SNPs and the time between seroconversion and AIDS-diagnosis or AIDS-related death.

## Results

**Figure 1:**
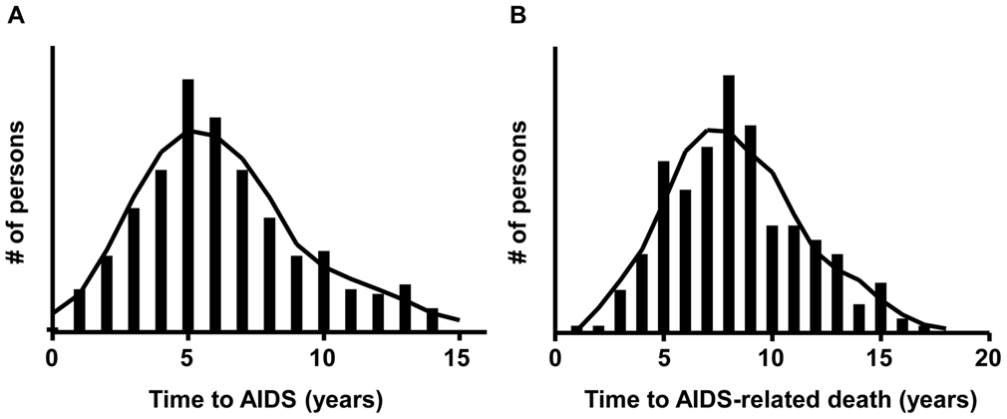
Distribution of the clinical course of HIV-1 infection in the ACS. Time from seroconversion to (A) AIDS-diagnosis or (B) AIDS-related death.

Time from seroconversion to AIDS-diagnosis or AIDS-related death was normally distributed in the ACS (Figure 1). To find host genetic markers that associate with disease progression after HIV-1 infection, we genotyped 455 samples with Illumina's Infinium HumanHap300 BeadChip which assays 317,503 SNPs [29]. After quality control (see Methods) and population stratification, association analysis was performed for 309,494 SNPs and HIV-1 disease course in 404 HIV-1 infected MSM and DU from the ACS using Cox regression survival analyses with AIDS according to the CDC 1993 definition [30] or AIDS-related death, as endpoints. The calculated λ values of 1.0231 and 1.0197 for the P-values of SNP associations with either AIDS-free survival or time to AIDS-related death, respectively, indicate that the remaining population stratification effect, after correction by using the two first eigenvectors as covariates, is minimal.

The top 10 associations between SNP genotypes and time to AIDS-diagnosis, had P-values smaller than 5.25×10^−5^, with P = 3.50×10^−6^ for the strongest statistical association (SNP rs1523635; Table 1). The top 10 associations between SNP genotypes and time to AIDS-related death had P-values smaller than 4.43×10^−5^, with P = 8.32×10^−6^ for the strongest statistical association (SNP rs7374396; Table 2). None of the associations between SNP genotypes and time to AIDS or AIDS-related death were genome-wide significant. However, the minor alleles of SNPs that ranked in the top 10 for association with time to AIDS or AIDS-related death were also associated with survival to other endpoints (Tables 1 and 2). None of the SNP genotypes identified to be associated with AIDS-diagnosis were associated with survival time after AIDS-diagnosis, indicating that their potential effect is at an earlier stage in the course of infection (Table 1).

**Table 1:** P-values for different endpoints in survival analysis and viral load set-point of top 10 SNPs associated in GWA study with time to AIDS-diagnosis.

						ACS					GRIV 2^nd^ phase	
SNP	CHR	Coordinate	Gene	Position	MAF	AIDS	CD4<400	Death	AIDS to Death	viral load set-point	NP vs Ctrl	RP vs CTRL
rs1523635	7	16982220	AGR3	90kb 3′	0.30	3.50E-06	ns	6.91E-05	ns	6.94E-03	2.42E-02	ns
rs4722020	7	21391068	SP4	40kb 5′	0.20	2.40E-05	8.29E-03	2.07E-03	ns	ns	ns	ns
rs6845554	4	9622271	SLC2A9	intron	0.40	2.90E-05	ns	2.36E-04	ns	ns	ns	2.75E-02
rs6827754	4	9627251	SLC2A9	intron	0.40	2.97E-05	ns	2.37E-04	ns	ns	ns	2.75E-02
rs2460000	1	2146222	SKI	5kb 5′	0.34	3.14E-05	9.72E-04	1.36E-04	ns	1.51E-04	ns	ns
rs4973374	2	231593659	SPATA3	30kb 5′	0.45	3.17E-05	1.80E-04	1.15E-05	ns	6.02E-03	ns	ns
rs3790623	1	37942165	CDCA8	intron	0.24	3.42E-05	5.12E-03	6.54E-04	ns	1.58E-02	ns	ns
rs1979738	15	31738020	RYR3	intron	0.31	3.74E-05	1.76E-02	1.01E-03	ns	ns	ns	ns
rs6018199	20	35443071	SRC	intron	0.14	5.11E-05	1.65E-03	3.07E-03	ns	ns	ns	4.22E-03
rs3753264	1	119711037	HAO2	5kb 5′	0.31	5.25E-05	2.22E-02	1.45E-03	ns	ns	ns	ns

ACS; Amsterdam Cohort Studies, GRIV; Genomics of Resistance to Immunodeficiency Virus cohort, CHR; chromosome, MAF; minor allele frequency, ns; not significant (P>5.00×10^−2^).

**Table 2:** P-values for different endpoints in survival analysis and viral load set-point of top 10 SNPs associated in GWA study with time to AIDS-related death.

						ACS					GRIV 2^nd^ phase	
SNP	CHR	Coordinate	Gene	Position	MAF	AIDS	CD4<400	Death	AIDS to Death	viral load set-point	NP vs Ctrl	RP vs CTRL
rs7374396	3	33645348	CLASP2	intron	0.27	8.32E-06	6.59E-05	4.62E-03	5.76E-03	4.45E-04	ns	ns
rs4485771	4	167112983	TLL1	intron	0.28	1.06E-05	2.59E-02	1.99E-04	ns	ns	ns	ns
rs4973374	2	231593659	SPATA3	30kb 5′	0.45	1.15E-05	1.80E-04	3.17E-05	ns	6.02E-03	ns	ns
rs2189915	7	80839680	Intergenic		0.32	1.61E-05	ns	4.40E-03	8.65E-04	9.89E-03	ns	ns
rs1434975	2	14722893	FAM84A:NSE1	25kb 3′	0.37	2.46E-05	ns	1.36E-02	ns	ns	ns	3.37E-02
rs3743833	16	10035456	GRIN2A	intron	0.13	3.71E-05	1.56E-03	5.56E-05	ns	6.41E-04	ns	ns
rs9379174	6	8167773	Intergenic		0.21	3.98E-05	ns	1.22E-02	ns	4.20E-02	ns	ns
rs1570023	20	60419644	C20orf151	intron	0.18	4.07E-05	2.44E-02	2.18E-02	4.56E-02	1.09E-02	ns	ns
rs541821	11	94801223	Intergenic		0.32	4.24E-05	2.98E-03	1.80E-02	ns	7.92E-03	ns	ns
rs2256688	10	44584427	Intergenic		0.30	4.43E-05	4.59E-02	2.96E-04	ns	ns	ns	ns

ACS; Amsterdam Cohort Studies, GRIV; Genomics of Resistance to Immunodeficiency Virus cohort, CHR; chromosome, MAF; minor allele frequency, ns; not significant (P>5.00×10^−2^).

The potential associations of the top 10 SNPs for both end points were then in a 2^nd^ phase analysis studied for confirmation in an independent and well-defined cohort of HIV-1-infected individuals. The Genomics of Resistance to Immunodeficiency Virus cohort (GRIV) is a French case-control study in which either rapid progressors or non-progressors were compared with HIV-1-negative controls [18], [20].

Three independent signals (four SNPs) that were associated with time to AIDS in our cohort had a P-value below 0.05 and a similar polarity in the GRIV cohort (Table 1). SNP rs1523635, which had the strongest association with a more rapid progression to AIDS in our cohort was also associated with rapid progression in the GRIV (P = 2.42×10^−2^). SNPs rs6845554 and rs6827754, which are in complete LD (r^2^ = 1) were both associated with rapid progression in the GRIV cohort as well (P = 2.75×10^−2^). Finally, SNP rs6018199 was associated with non-progression in the ACS cohort as well as in the GRIV cohort (P = 4.22×10^−3^).

Of the top 10 associations between SNP genotypes and time between seroconversion and AIDS-related death, the minor allele of SNP rs1434975 was associated with rapid progression in the ACS and in the GRIV cohort on extremes in disease progression (P = 3.37×10^−2^; Table 2).

## Discussion

We identified novel genetic loci that may be associated with the clinical course of HIV-1 infection in the ACS. None of the associations between SNPs and time to AIDS or time to AIDS-related death that ranked in the top 10, were genome-wide significant. Interestingly, the majority of the SNPs involved were also associated with other phenotypes that are related to HIV-1 infection, such as set-point viral load, although one could argue that those are not independent phenotypes. Furthermore, three independent signals that ranked in the top 10 for associations with time to AIDS, and one SNP from the top 10 associations with time to AIDS-related death, were also associated with disease course in the GRIV cohort of HIV-1 infected individuals, with the same effect of the minor allele on disease progression. This independent confirmation is evidence for an actual association between the identified gene regions and HIV-1 disease course, and not a statistical artifact.

The four gene regions that were found to be associated with disease progression in both ACS and GRIV are distributed around the genome. SNP rs1523635 is located 90 kb downstream of Anterior Gradient Homolog 3 (AGR3), alias Breast Cancer Membrane Protein 11 (BCMP11). Interestingly, AGR3 is known to interact with LY6/PLAUR Domain Containing 3 (LYPD3), and the LY6 family has been associated with HIV-1 disease progression although the mechanism is unknown [31]. SNPs rs6845554 and rs6827754, which are in high LD, are both located intronic of Solute Carrier Family 2, Member 9 Protein (SLC2A9). A potential effect of this gene on the clinical course of HIV-1 infection has not been reported. SNP rs6018199 is located in an intron of the Proto-Oncogene Tyrosine-Protein Kinase (SRC). The HIV-1 *Nef* gene was found to selectively activate certain members of the SRC kinase family, including SRC itself, which may contribute to HIV-1-associated nephropathy which is the most common cause of chronic renal failure in HIV-1-infected individuals [32].

The SNP that was found to be associated with time to AIDS-related death in the ACS and associated with disease progression in the GRIV is SNP rs1434975. This SNP is located 25 kb downstream of the gene Family with Sequence Similarity 84, Member A (FAM84A). The function of the gene is largely unknown and interaction with HIV-1 has not been reported.

None of the top 10 SNPs identified in our present study map to gene regions that were identified in previous GWAS on HIV-1 control that mainly identified associations between HIV-1 control and SNPs on chromosome 6 [14], [15], [18]–[20]. In a previous study, we were able to confirm SNP rs2395029 in the HCP5 gene region, in complete linkage with HLA-B57, and SNP rs9264942 upstream of the HLA-C gene, that were identified by Fellay et al [15] to be associated with viral load set-point and/or CD4^+^ T-cell depletion. In our GWAS, however, these associations did not have genome-wide significant P-values [16]. Moreover, HLA-B57 and HLA-B27 were also only associated with time from seroconversion until AIDS-diagnosis but not with time between AIDS-diagnosis and AIDS-related death [6], [16]. Differences in ancestry, gender, transmission route, study design (case-control versus survival analysis), phenotype used for association, or other yet unknown factors may explain the variable outcome of different studies. Indeed, two studies that also used survival time as a phenotype identified signals outside the chromosome 6 HLA-gene region as well [21], [23]. This illustrates that different end-points should be used to unravel the full spectrum of host genetic factors that may be associated with the clinical course of the infection.

The lack of confirmation of SNP associations with phenotypes related to HIV-1 disease may point to false-positive associations. However, it cannot be excluded that lack of confirmation relates to the calendar time of seroconversion of the cohort participants in combination with the adaptation of HIV-1 to its host environment Indeed, certain associations between host genetic background and the clinical course of infection or viral load set-point have disappeared over time at a population level [33], [34] (van Manen et al, submitted for publication).

GWAS may reveal genes that can be considered new targets for drug development. In addition, SNPs strongly associated with HIV-1 disease progression, like the SNPs identified in this study, may be applied in routine diagnostics to predict disease course, or to more accurately estimate if antiviral therapy should be initiated. Indeed, screening for polymorphisms in the human genome that influence the course of infection may ultimately allow for a better fine-tuning of patient management.

## Materials and Methods

### Study population

We studied HIV-1 infected men who have sex with men (MSM) and drug users (DU) who participate in the ACS on HIV infection and AIDS. The HIV-infected MSM were enrolled in the cohort between October 1984 and March 1986 [35]. In the first serum sample taken at entry in the cohort, 239 men tested positive for HIV antibodies (recombinant HIV-1/-2 enzyme immunoassay (Abbott, Chicago, Illinois) with an HIV-1 Western blot IgG assay (version 1.2, Diagnostic Biotechnology Ltd., Singapore) for confirmation; five of these men refused to participate further. Of the HIV-1 negative men, 131 subsequently seroconverted during active follow-up (until May 1996). For seroprevalent individuals, an imputed seroconversion date (on average 18 months before entry into the ACS) was used [36]. Seroprevalent individuals and prospective seroconverters were studied as one group [11], [16]. Most seropositive men (n = 243 [67%]) did not receive any early treatment, 70 (19%) received zidovudine monotherapy, 10 (3%) received didanosine monotherapy, and 42 (11%) received other ineffective antiretroviral therapy before AIDS-diagnosis. The mean age of participants at the time of (imputed) seroconversion was 34.5 years (range 19.5–57.7 years). A DNA sample was available from 335 of these 365 cohort participants for genotyping analysis (205 seroprevalent cases and 130 seroconverters). This cohort of MSM has been used for confirmation of host genetic factors and disease course [37]–[40].

The participants of the ACS on HIV and AIDS among DU started enrolment in 1985 [41]. Asymptomatic men and women who were living in the Amsterdam area and who reported intravenous drug use in the preceding 6 months were enrolled in a prospective study on the prevalence and incidence of HIV-1 infection and risk factors for AIDS. We included 83 individuals who seroconverted for HIV antibodies during active follow-up. At entry in the cohort study 37 individuals were positive for HIV antibodies but had a negative test result before entry (i.e., retrospective seroconverters). For all 120 HIV-1 infected DU (76 men and 44 women) used for our present analysis, the imputed seroconversion date was calculated and used for further analysis [42]. Mean survival time was similar for male and female participants of the DU cohort and for the DU versus MSM cohorts. Most seropositive DU (n = 69 [58%]) did not receive any early treatment, 28 (23%) received zidovudine monotherapy, and 23 (19%) received other ineffective antiretroviral therapy before AIDS-diagnosis. The mean age of DU participants at the time of (imputed) seroconversion was 31.5 years (range 19.1–55.9 years). A DNA sample was available from all 120 DU participants, hence all were included in further analyses.

The ACS has been conducted in accordance with the ethical principles set out in the declaration of Helsinki and all participants provided written informed consent. The study was approved by the Academic Medical Center institutional Medical Ethics Committee of the University of Amsterdam.

### Genome-wide association scan genotyping

Genotyping was performed according to the Infinium II protocol from Illumina (Illumina, San Diego, USA) [29]. Samples were only included if a minimum 95% call rate was observed for the sample in BeadStudio 3.1 data analysis software (Illumina Inc, San Diego, USA). Three samples that had a call rate below 95% were repeated using a new DNA aliquot and analyzed successfully.

### GWA quality control and linkage disequilibrium

SNPs were excluded from analysis for the following reasons (applied in order): call rate <95% (n = 1910), allele frequency <1% in (n = 280) and deviation from Hardy Weinberg equilibrium (P<0.0001, n = 5819). Subsequent analyses were performed on a dataset of 309,494 SNPs.

LD was characterized using WGA Viewer [43]. WGA Viewer was also used for creation of genome-wide overview plots, Q-Q plots, and calculation of the genomic inflation factor λ.

### Identification of population stratification

Eigenstrat, implemented in Eigensoft, was used to identify outliers in the population who were subsequently removed from the analysis. The first two eigenvectors were used as covariates to correct for any remaining population structure. As a result, genetic data from 404 individuals (304 MSM and 100 DU) were used for further analysis.

### Phenotype

AIDS according to the Centers for Disease Control (CDC) 1993 definition [30] was used as an endpoint in survival analysis. The other endpoint used in survival analysis was AIDS-related death, defined as death with AIDS-related malignancy, death with AIDS-opportunistic infections, or death with AIDS-related cause not specified by the treating physician.

### Virological assays

Samples collected prior to 1997 were tested using nucleic acid sequence-based amplification (NASBA) HIV-1 RNA QT (NASBA, bioMerieux, Boxtel, Netherlands). Samples with a viral load below the quantification threshold of the NASBA assay (1,000 copies/ml, n = 68), were re-tested in 2008 using the RealTime HIV-1 assay (Abbott Laboratories, Abbott Park, IL) with a threshold of 40 copies/ml. Viral load data were analyzed after log_10_ transformation. Viral load set-point was defined as the relatively steady level of HIV-1 RNA at 18–24 months after seroconversion. Set-point viral load data were available for 385 (301 MSM and 84 DU) participants.

### Statistical analyses

Cox regression survival analysis was used to analyze genome-wide associations for all SNP genotypes in a genotypic model and the course of HIV-1 infection, using AIDS and AIDS-related death as an endpoint. SNPs with less than 8 individuals homozygous for the minor allele were excluded from the analysis (n = 74,967). Cox proportional-hazards regressions and corresponding P-values were computed with ProbABEL v0.1-3 software [44] which is based on the R library ‘survival’ developed by Thomas Lumley (function coxfit2). The association of the SNP with viral load set-point was tested using one-way analysis of variance (ANOVA). All statistical analysis were performed using GraphPad Prism 5.0 software (GraphPad Software, San Diego, CA, USA), and the R package (http://www.r-project.org).

### Confirmation study population

The Genomics of Resistance to Immunodeficiency Virus (GRIV) cohort was established in France in 1995 to generate a large collection of DNA for genetic studies to identify host genes associated with either rapid progression (n = 85) or non-progression (n = 275) to AIDS. Further information on participants, genotyping, quality control and statistical analysis are described in Limou et al [18] and Le Clerc et al [20].
